# Cancer Immunotherapy: Historical Perspective of a Clinical Revolution and Emerging Preclinical Animal Models

**DOI:** 10.3389/fimmu.2017.00829

**Published:** 2017-08-02

**Authors:** William K. Decker, Rodrigo F. da Silva, Mayra H. Sanabria, Laura S. Angelo, Fernando Guimarães, Bryan M. Burt, Farrah Kheradmand, Silke Paust

**Affiliations:** ^1^Department of Pathology and Immunology, Baylor College of Medicine, Houston, TX, United States; ^2^Dan L Duncan Cancer Center, Texas Children’s Hospital, Houston, TX, United States; ^3^Center for Cell and Gene Therapy, Baylor College of Medicine, Houston, TX, United States; ^4^Center for Human Immunobiology, Department of Pediatrics, Texas Children’s Hospital, Houston, TX, United States; ^5^Women’s Hospital – CAISM, University of Campinas, Campinas, Brazil; ^6^Diana Helis Henry Medical Research Foundation, New Orleans, LA, United States; ^7^Michael E. DeBakey Department of Surgery, Division of Thoracic Surgery, Baylor College of Medicine, Houston, TX, United States; ^8^Department of Medicine, Pulmonary and Critical Care, Baylor College of Medicine, Houston, TX, United States

**Keywords:** history of immunotherapy, canine cancer models, patient-derived xenograft models, mouse models of cancer, checkpoint blockade, tumor immune evasion

## Abstract

At the turn of the last century, the emerging field of medical oncology chose a cytotoxic approach to cancer therapy over an immune-centered approach at a time when evidence in support of either paradigm did not yet exist. Today, nearly 120 years of data have established that (a) even the best cytotoxic regimens only infrequently cure late-stage malignancy and (b) strategies that supplement and augment existing antitumor immune responses offer the greatest opportunities to potentiate durable remission in cancer. Despite widespread acceptance of these paradigms today, the ability of the immune system to recognize and fight cancer was a highly controversial topic for much of the twentieth century. Why this modern paradigmatic mainstay should have been both dubious and controversial for such an extended period is a topic of considerable interest that merits candid discussion. Herein, we review the literature to identify and describe the watershed events that ultimately led to the acceptance of immunotherapy as a viable regimen for the treatment of neoplastic malignancy. In addition to noting important clinical discoveries, we also focus on research milestones and the development of critical model systems in rodents and dogs including the advanced modeling techniques that allowed development of patient-derived xenografts. Together, their use will further our understanding of cancer biology and tumor immunology, allow for a speedier assessment of the efficacy and safety of novel approaches, and ultimately provide a faster bench to beside transition.

## A Brief History of Cancer Immunotherapy

Perhaps no innovation has had a more meaningful impact upon modern medicine than the development of vaccination (1), a revolutionary achievement accomplished in piecemeal fashion over the course of the 18th and 19th centuries. Though prevention of smallpox by purposeful inoculation with *variola minor* may have had origins as ancient as China’s third century BC Qin dynasty (2), it is clear that the Ottoman Turks were commonly employing this practice by 1718, when the wife of the British ambassador to Istanbul, Lady Mary Wortley Montague, observed the local custom of variolation and popularized it upon her return to England (3, 4). While variolation, or infection with *variola minor*, was not a perfect vaccination strategy, its case fatality rate of 1–2% was far lower than that of fulminant *variola major* infection, a malady that killed up to 30% of its victims. Some years after variolation became entrenched among the eighteenth century European medical establishment, reports regarding possible protective efficacy of cowpox infection began to circulate in the literature. The earliest known such report may be credited to British physician Dr. John Fewster whose paper, “Cowpox and its Ability to Prevent Smallpox,” was read to the London Medical Society in 1765. By 1796, Royal Society Fellow Edward Jenner had demonstrated that the protective immunity of cowpox could be passed between vaccinees and most importantly, that 23 inoculated individuals were genuinely immune to smallpox as evidenced by failure to become symptomatic after variolation (5).

Following Jenner’s revolutionary discovery, nearly a century elapsed before the development of a second vaccine. Unquestionably, Jenner had been extraordinarily lucky in his discovery of a natural pre-attenuated pathogen, a vaccine strain as perfect as any generated by years of serial passage in a laboratory. Subsequent development of future vaccines would need to wait for Antonio Bassi to formally propose the germ theory of disease in 1844 (6), for Louis Pasteur (7) and Joseph Lister (8) to publish evidence in support of this revolutionary theory, and finally for Robert Koch to develop his infectious disease postulates between the years 1884 and 1890 (9). In the century that separated Jenner and Koch, medical science proved that pathogens were the causative agents of disease and became aware that weakened or killed pathogens could often provoke protective immunity in inoculated hosts. This highly conducive scientific environment ushered the development of a broad array of vaccines in the late 19th and early 20th centuries. Beginning with the successful demonstration of the Pasteur/Roux rabies vaccine in 1885, eight important vaccines were introduced, providing substantial protection against the effects of ancient killers like plague (1897), cholera (1917), and typhoid (1917); as well as more the more contemporary scourges of diphtheria (1923), pertussis (1926), tuberculosis (1927), and tetanus (1927). Efforts continued into the twentieth century with the development of vaccines against viral diseases including yellow fever (1935), influenza (1945), polio (1955), measles (1963), mumps (1967), and rubella (1969). In the 1980s, advances in immunology, molecular biology, and medicinal chemistry led to the generation of multivalent cell-free polysaccharide vaccines for the prevention of meningococcal meningitis (Menomune, 1981) and pneumococcal pneumonia (PneumoVax, 1983). In late 1981, the first vaccine based upon a single purified surface antigen (HBsAg) became available for the prevention of hepatitis B (HBV) (10, 11). At this moment of scientific triumph, the ability of medical science to manipulate the human immune system appeared unparalleled, and additional contemporaneous discoveries engendered optimism that immune-mediated therapies might be used to treat or even to cure cancer (12, 13).

Though the idea of using the immune system to fight neoplastic disease was novel in the 1980s, its practice was not. William B. Coley, a nineteenth century surgeon at the Hospital for the Ruptured and Crippled (now the Hospital for Special Surgery), developed the first immune-based treatment for cancer at the end of the nineteenth century. Deeply affected by the death of his first patient from metastatic sarcoma, Dr. Coley ignored the siren song of complacency and instead embraced the stubborn recalcitrance that defines all revolutionaries. He delved deeply into the eighteenth century medical literature and unearthed 47 case reports in which concomitant infection seemed to have caused the remission of an otherwise incurable neoplastic malignancy. Most striking to Dr. Coley was an apparent connection between erysipelas, a streptococcal infection of the dermis, and the remission of soft tissue sarcomas. When Dr. Coley began injecting his cancer patients with the *S. pyogenes* causative agent of erysipelas, he encountered a surprising impediment. He discovered that it was very difficult to induce erysipelas in most patients and also exceptionally difficult to cure among the few in which productive infection was established. Two patients even died from disseminated septicemia rather than their underlying cancers. In response, Coley settled upon a non-infectious admixture of heat-killed *S. pyogenes* and heat-killed *B. prodigious* (now reclassified as *S. marcecsens*). This fortuitous combination of Gram-positive and Gram-negative bacteria possessed a wide array of immunostimulatory properties that allowed Dr. Coley to achieve excellent long-term cure rates that in some instances remain unrivaled by medical science in the 81 years since his death (14–18). Despite impressive clinical results first published in 1893, Dr. Coley was viewed with suspicion by the medical establishment of the day; and while Paul Ehrlich would propose the cancer immunosurveillance hypothesis only 16 years later (19), contemporaries didn’t make a connection between “Coley fluid” and the nascent science of immunology. Therefore, in his own time, the lack of a suitable explanation for Coley’s results ultimately doomed his treatment regimen, and it is the century-long search for mechanism that has come to define William Coley’s legacy (20). His initial observations have in large part led to the discovery of the soluble signaling factors that modulate immune function, the pattern recognition receptors responsible for the detection of infectious organisms (21–24), and the state-of-the-art checkpoint inhibitors that have become the mainstay of modern immuno-oncology (25–27).

Yet, despite the outsized role that Coley’s discoveries ultimately played, little happened in the field between Coley’s death in 1936 and the advent of immunology’s modern era some two decades later. This era reasonably began in 1957 with the discovery of interferon by Isaacs and Lindenmann (28, 29) as well as the founding of the Cancer Research Institute of New York, dedicated to the development of immune-based treatments for cancer, by William Coley’s daughter, Helen Coley Nauts. In 1959, the husband and wife team of Ruth and John Graham published the first ever cancer vaccine study, a 114 patient cohort of gynecologic cancer patients treated with adjuvanted tumor lysate (30). Despite a 22% incidence of remission or stable disease, the work went largely unnoticed. While the mechanism by which the Graham vaccine exerted any efficacy is unknown, the contemporaneous assumption invoked the generation of tumor-specific antibody—the only known or suspected adaptive mechanism in the mid-1950s. The existence of T-cells and the critical role of the cellular immune response in adaptive immunity would not be fully characterized until Jacques Miller’s seminal publication in 1967 (31). Immediately after, a number of other crucial discoveries that would set the stage for the advent of cancer immunotherapy were made in rapid-fire succession. These included the discovery and characterization of dendritic cells by Ralph Steinman in 1973 (32), Zinkernagel and Doherty’s description of MHC restriction in 1974 (33), and Eva Klein’s documentation of natural killer (NK) cell activity in 1975 (34, 35). In conjunction with the simultaneous revolution in molecular biology that accompanied isolation and characterization of the first restriction endonuclease in 1970 (36), the initial immune-based cancer treatments began to make their way into clinical medicine even though, as in William Coley’s time, the immunologic component of these regimens was not understood. Bone marrow transplantation for the treatment of hematologic malignancies was pioneered in the mid-1970s at the University of Minnesota, while molecular cloning of the interferon gene and subsequent industrial-scale production permitted Talpaz and colleagues at the MD Anderson Cancer Center to begin treating chronic myeloid leukemia patients with recombinant interferon alpha (37). Nonetheless, the idea that the immune system could play an important role in the treatment of many cancers still remained a concept solidly external to the purview of mainstream oncology. Clinical oncologists didn’t yet buy what researchers were selling, and even if they had wanted to, there was no real product to give to their patients.

Much needed clarification came from the works of Schreiber and colleagues, who in 1998 and 2001, provided key evidence of T cell-mediated tumor-specific immune surveillance, *bona fide* antitumor immune responses, and evidence of tumor immune escape. This work made clear that lymphocyte and IFN-γ-mediated effector functions collaborate to protect against the development of carcinogen-induced cancers, and that over time, immune pressure on tumors selects for tumor cells with reduced immunogenicity (38, 39). Thus, on one hand, the immune response is effective as a tumor-suppressor, while on the other, immune pressure leads to the selection of tumor cells that subsequently escape eradication by immune-mediated mechanisms and thus still survive in an immune-competent host. This work in part explained the apparent paradox of tumor formation in immunologically intact individuals and laid the groundwork for the discovery of tumor-induced immune exhaustion pathways.

It was ultimately two watershed events in 2010 and a third in 2011 that forced acceptance of immuno-oncology onto ambivalent clinical practitioners; however, these unrelated events were each several decades in the making. Though cytotoxic T-lymphocyte antigen 4 (CTLA-4) was first identified by Brunet and colleagues in 1987 (40), its function as a critical immune checkpoint remained obscure until published by Jim Allison’s group in 1995 (41), and the potential for treating cancer by its blockade unappreciated until the following year (42). Fourteen more years would then elapse from the time that these preclinical data were published to the time that the definitive clinical study permitted FDA approval of the revolutionary checkpoint inhibitor ipilimumab for the treatment of stage IV melanoma (25). In that same year, 20 years of work and three phase III clinical trials finally resulted in FDA approval of sipuleucel-T, a *bona fide* dendritic cell vaccine, for the treatment of stage IV metastatic but asymptomatic castrate-resistant prostate cancer (43). With excitement in immuno-oncology suddenly at a fevered pitch, the bar was improbably raised the following year by the unexpected and stunning success of a genetically modified T-cell strategy many thought would never work. First described experimentally in 1993 (44), the chimeric antigen receptor (CAR) strategy that linked a tumor antigen-specific single-chain immunoglobulin variable region (scFv) to CD3-ζ and/or other costimulatory signaling domains like CD28 seemed too clever by half and had performed abysmally in early clinical studies (45, 46). Yet, replacement of the CD28 signaling domain with that of 4-1BB by Carl June and colleagues resulted in a complete and durable remission of a pediatric patient with treatment-refractory chronic lymphocytic leukemia following adoptive transfer of construct-transduced autologous T-cells (47). Over the next 5 years, the field continued to build on these successes with further breakthroughs in CAR T-cell therapy including application to additional diseases (48, 49), novel target validation, and the addition of suicide safety switch technologies (50). By the end of 2016, four different checkpoint inhibitor drugs blocking two different pathways (25–27, 51) had received FDA approval for the treatment of melanoma, renal cell carcinoma (RCC), lung cancer, lymphoma, and cancers of the bladder. Additional approvals for squamous cell head and neck cancers seem all but certain given the recent publication of promising data (52).

## Targeting Immunosuppression

Many tumor microenvironments, such as lymphoma (53) and lung carcinoma (54) are enriched in immune suppressive cells, such as regulatory T cells, myeloid-derived suppressor cells (MDSCs), or type 2 macrophages (M2), all of which contribute to immune exhaustion *via* the expression of inhibitory ligands, suppressive cytokines, and tumor-promoting factors (55). It is, therefore, not surprising that high numbers of tumor-resident regulatory T cells, M2 macrophages, and/or MDSC are correlated with poor outcomes and with advanced stages of cancer (56–58). Thus, therapies that reduce the induction, recruitment, or immune suppressive activities of these immune suppressive cells, or lead to their deaths, have been explored as cancer immunotherapies that specifically target the mechanisms of immune suppression that allow tumor escape. Specifically, receptor tyrosine kinase (RTK) inhibitors have been explored as cancer immunotherapy drugs, as they target growth factor-mediated signaling pathways and reduce angiogenesis, survival, proliferation, and metastasis formation of tumors (59). Two of these, sunitinib and sorafenib, target signaling by vascular endothelial growth factors, platelet-derived growth factor receptor alpha/beta (PDGFRα/β), and stem cell growth factor receptor signaling (60). Sunitinib also targets the RTK Flt-3 and the serine/threonine-specific protein kinase Raf, while sorafenib also targets signaling through the RTK c-RET. Both sunitinib and sorafenib can reduce the frequency of regulatory T cells in mouse models of cancer and in patients with metastatic renal cell carcinoma (RCC) (61). Similarly, sorafenib-reduced regulatory T-cell frequencies in patients with hepatocellular carcinoma. While the mechanisms for regulatory T-cell reduction are unclear, treatment did allow the development of some host immunity against the tumor (61). MDSCs are also modulated by RTK, and treatment with sunitinib has been shown to reduce the proliferation of monocytic MDSC while inducing cell death by apoptosis in granular MDSC in mouse models of cancer, and the frequency of both subsets of MDSC was reduced in patients with metastatic RCC (61). Treatment benefits correlated with an increase in both CD4^+^ and CD8^+^ tumor-infiltrating T-cells even though sunitinib and sorafenib impact T-cell effector functions differently. While treatment with sunitinib enhances IFN-γ production and cytolytic antitumor activity of CD8^+^ cytotoxic T-cells, treatment with sorafenib reduces the expression of T-cell expressed activation markers (CD25 and CD69) and results in reduced IL-2 production and increased T-cell apoptosis upon *in vitro* stimulation of T cells with the lectin phytohemagglutinin (62, 63). How sunitinib and sorafenib modulate T-cell activity in cancer patients is currently not well understood and requires further investigation.

The effects of sunitinib and sorafenib on NK cell biology and antitumor responses have also been investigated. While Sunitinib treatments did not alter the frequency of peripheral blood NK cells in patients suffering from metastatic RCC, both sunitinib and sorafenib induced NKG2D ligand expression on nasopharyngeal and hepatocellular carcinoma cell lines, which correlated with increased triggering of NK cell-mediated lysis (64). Altogether, the targeting of growth factor signaling pathways has effects on angiogenesis, cell proliferation, survival, and host immunity to tumor. Combination therapies are currently being explored to combine the beneficial effects of RTK with other immune modulatory approaches including cytokine administration and checkpoint blockade. The combined effects of these on the host response to tumor remain to be evaluated.

While the targeting of immunosuppressive cell populations is a promising clinical strategy, antagonism of the soluble cytokines and other suppressive factors released by these cell types is also being considered as a strategy to augment potentiation of antitumor responses. In the development of this strategy, oncology borrows from the rich tradition of clinical rheumatology that has produced dozens of clinically approved cytokine and cytokine receptor antagonists for the treatment of autoimmune conditions. These include a wide variety of TNF-α blockers (e.g., infliximab, etanercept, and adalimumab among others) for the treatment of many different autoimmune conditions including Crohn’s disease, ulcerative colitis, psoriasis, rheumatoid arthritis, and ankylosing spondylitis (65); the IL-1 receptor antagonist anakinra approved for the treatment of rheumatoid arthritis (66) and moving through late stage clinical trials for the treatment of other autoimmune conditions; IL-4/IL-13 receptor antagonists (lebrikizumab, pitrakinra, dupilumab, tralokinumab) currently in clinical trials for the treatment of asthma (67, 68), and IL-2 receptor antagonists used to treat multiple sclerosis (daclizumab) (69) and prevent transplant rejection (basiliximab) (70). Efforts at aimed at direct blocking of IL-10 signaling, while promising in experimental model systems, remain in preclinical stages of development at present (71). In contrast, there exist an enormous number of peptides, anti-sense oligonucleotides, monoclonal antibodies, and small molecule inhibitor drugs in various stages of clinical development that are designed to block TGF-β signaling in a variety of different ways (72, 73). Galunisertib, a small molecule inhibitor of the TβRI-associated kinase, is in clinical trials for a number of different neoplastic indications (74–76) as is fresolimumab, a monoclonal antibody that blocks receptor ligand interaction of all TGF-β isoforms (77). Trabedersen is a novel TGF-β-targeting antisense oligonucleotide drug moving through the clinical development pipeline for the treatment of malignant glioma (78). In addition, immuno-oncology has borrowed the IL-6 inhibitor tocilizumab, already approved for the treatment of rheumatoid arthritis (79) and other conditions, to effectively neutralize the cytokine storm invariably triggered by successful CAR T-cell administration (80).

## Modeling Vaccine Immunotherapy: Unmet Needs

While development of checkpoint inhibitor therapies and transgenic CAR strategies continues to progress, analogous progress in vaccine immunotherapy has lagged significantly by comparison despite first mover advantage in both theory and practice. The success of the former partially explains the failure of the latter. Checkpoint inhibitor drugs and CAR T-cells do not just extend lives by weeks or months, i.e., are not just prolonging the inevitable while the patient still suffers and eventually dies. Instead, in certain subsets of patients, the new immune-based strategies offer what appears to be a permanent and durable cure. A decade ago, stage IV melanoma was a death sentence, whereas today, up to half of all stage IV melanoma patients can expect to be cured of their disease through combination anti-CTLA-4 (ipilimumab)/anti-PD-1 (nivolumab or pembrolizumab) checkpoint inhibition (Table 1) (81–83). These results stand in sharp contrast to those obtained following administration of the sipuleucel-T putative DC vaccine. Patients administered that this very expensive treatment regimen exhibit only an extra 4 months of OS with no concomitant enhancement of the long tail on the right side of the Kaplan–Meier survival curve. By 5 years post-administration, patients administered sipuleucel-T exhibit a survival probability identical to that of those who receive placebo (43). Sipuleucel-T ultimately doesn’t cure anyone, and these disappointing results are roughly on par with those of over 400 other dendritic cell vaccine trials carried out between 1995 (84) and the end of the last decade (85, 86), the time at which interest in such trials significantly waned in response to the success of checkpoint inhibition. Further, other high profile vaccine strategies based on 1990s technology also performed exceptionally poorly in the clinic. Two large phase III clinical trials based on the GVAX vaccination platform (irradiated, allogeneic cancer cell lines transduced to express high levels of GM-CSF) (87) were terminated early when interim futility analyses indicated that patients who had received the vaccine were actually dying faster than those in the control arms (88, 89). Several other highly touted vaccine strategies including Melacine, CanVaxin, OncoPhage, Theratope, Bec2, and TRICOM (ProstVac and PanVac) also failed in equally definitive fashion in phase III clinical studies of their own (90). Given the sheer amount of positive preclinical and early clinical data required to justify the expense and risk of a phase III trial, how could this possibly have happened? Why did immuno-oncology vaccine therapies in particular perform favorably in model systems yet so poorly in real-world studies? The answers to these somewhat perplexing questions actually lie in the perceived experimental strengths of the therapeutic models systems.

**Table 1:** FDA approved checkpoint inhibitors targeting the programmed death or cytotoxic T lymphocyte antigen-4 pathways.

Drug and trade name	Target	FDA approval
Prembrolizumab KEYTRUDA	PD-1	Advanced melanoma PDL-1-positive metastatic non-small cell lung cancer Advanced or metastatic urothelial carcinoma Refractory classical Hodgkin’s lymphoma Recurrent or metastatic head and neck squamous cell carcinoma
Nivolumab OPDIVO	PD-1	Metastatic melanoma Locally advanced or metastatic urothelial carcinoma PDL-1+ non-small cell lung cancer (NSCLC)
Avelumab BAVENICO	PDL-1	Metastatic merkel cell carcinoma
Durvalumab IMFINZI	PDL-1	Metastatic bladder cancer
Atezolizumab TECENTRIQ	PDL-1	Locally advanced or metastatic urothelial carcinoma, PDL-1+ NSCLC
Ipilimumab YERVOY	Cytotoxic T-lymphocyte antigen 4	Metastatic melanoma

## Mouse Models of Cancer

The development of inbred animal mouse strains in the early twentieth century allowed researchers to perform basic experimentation without the confounding influence of biologic or (in the case of immunology) antigenic variation. Further refinements in husbandry also permitted model systems to be free of many confounding environmental variables as well. Subsequently, advanced techniques in genetic engineering allowed gain- and loss-of-function mutations in cancer to be studied in isolated and controlled genomic environments.

Lung cancer, the leading cause of cancer-related death in the world (91) is ideally poised for development of effective tumor vaccines that could significantly impact cancer survival. Lung cancer is divided into two major histopathological groups: small cell carcinoma (SCC) and non-small cell lung cancer (NSCLC), where the latter makes up nearly 85% of all lung cancer incidences. Histologically, NSCLC is further subdivided into adeno- (40–50%), squamous- (25–30%), and large- (10–15%) cell carcinomas. Notably, SCC, which comprise only 10–15% of all lung cancers, are highly aggressive, and have significantly reduced overall survival when compared to NSCLC. In addition, SCC arise from neuroendocrine cells within the lung, remain poorly studied because they are not usually amenable to surgical curative resection, and lack animal models that can closely recapitulate their characteristic. Because of their similarities in histopathology and tumor progression stages between mouse and human NSCLC, several useful lung cancer models have been developed to evaluate potential therapies (92, 93). Initial models of lung cancer relied on the application of chemical carcinogens [benzo(α)pyrene or 3-methylcholanthrene] to either directly the trachea or by skin painting, and it is likely that technical difficulties in these studies resulted in poor reproducibility of data (94–97). Further, success to induce mice by skin paining with another chemical carcinogen, *N*-nitroso-trischloroethylurea, was found to be strain dependent (98–102).

Inactivation of tumor-suppressor genes *PTEN, SMAD4*, and *p53* (103) and mutations or amplifications of oncogenic genes *Kras, EGFR*, and *ERBB2* (104), have been linked to human lung cancer. Because *PTEN* is often highly dysregulated, it is now recognized as a prognostic marker in human lung cancer (105). Based on these findings, several mouse models of lung cancer have been developed through genetic mutation of these pathways. For example, mice that lack *Pten* in airway epithelia develop hyperplasia, whereas concurrent ablation of transcription factor *Smad4* results in spontaneous development of adenosquamous lung cancer in the proximal bronchi beginning at approximately 7–9 months of age (106). At 12 months of age, 100% of mice deficient in airway epithelial Smad4 and Pten develop lung cancer with nearly 2/3 show distant metastasis to the stomach, liver, and spleen. Similarly, loss of *Lkb* and *Pten* using adenovirus-induced cre-recombinase resulted in lung cancer with features of squamous lung cancer (107). Further, because airway-specific targeted deletion of tumor suppressor genes result in predictable lung tumors that spontaneously metastasize, they provide excellent preclinical models to examine the role of innate and acquired immune responses to tumor, as well as *in vivo* biological studies to examine tumor latency.

These critical developments generated robust experimental systems in which basic biological questions were carefully asked and answered; however, it is important to also consider that the sterile environment of experimental rigor may prove to be ineffectual when tasked with duplicating the complexities of multifactorial neoplastic disease. Here, the lack of genetic, antigenic, and environmental variability can limit the usefulness of experimental systems co-opted by immuno-oncology for use as preclinical therapeutic models (108). Even further, the clean environments in which experimental animals are housed may prove detrimental to modeling real-world interactions between cancer and the immune system as the lack of exposure to pathogens and normal commensals has been shown to impair natural immune maturation and development (109). Hence, treatments tailored to the activation of poorly physiologic immune systems have been tested in uniform and non-variant disease models that bear too little resemblance to human cancers, which may help explain why some of these cancers can overpower putative experimental therapies.

## Canine Models of Cancer and Their Usefulness in Immunotherapy Development

While the criticisms of rodent therapeutic models are many and significant, this is not to suggest that these models should be discarded. On the contrary, the cost, reproducibility, well-characterized attributes, and wide biologic variation of these model systems still render them extremely valuable for proof-of-concept studies. However, once such studies are completed and promising results obtained, validation should next proceed using a physiologic real-world model system before advancing to human clinical trials. For an increasing number of investigators, the companion domestic canine population is ably serving as the bridge between rodents and humans. Cancer is the most common cause of death in companion canines, impacting over four million animals per year in the United States (110, 111); and the aspects of this model that confound the basic researcher are the precise attributes in which the translational researcher should delight.

1. The model is spontaneous. Canine cancers develop in response to real-world environmental and genetic stimuli, and subsequently evolve in response to real-world immunologic selection pressure. Unlike spontaneous rodent models in which an investigator might wait months or years for the development of disease, brand new canine cases are available weekly or even daily at veterinary oncology clinics serving the largest metropolitan areas.
2. The model is outbred. Treatments may be modeled in a background of high genetic variability that mimics that of human populations.
3. Canine immune systems are physiologically similar to those of humans. Companion canines have been raised in the same environments as their human masters, sharing a broad array of pathogens and commensals as well as typical regulatory T-cell responses that accompany mature immune development in response to a lifetime of unpredictable and variegated stimuli (112–114).
4. Even the treatment environments are heterogeneous. Companion animals are cared for in the clinic by real-world veterinarians who consider factors such as convenience, cost, and feasibility. Animals are cared for holistically so that the disease is not treated at the expense of all other considerations. Rarely are such realities taken into account by graduate and postdoctoral researchers caring for rodents. Even more importantly, canine patients come with owners, some accommodating and some cantankerous, tasked with continuing important aspects of treatment on an outpatient basis. In this regard, domestic canines also permit modeling of real-world compliance issues that can doom any therapeutic regimen if the treatment becomes too inconvenient or onerous.

Given these advantages, it is unsurprising that the only USDA or FDA approved cancer vaccine in the United States, Oncept (xenogenic tyrosinase DNA for the treatment of oral melanoma), is licensed for use in dogs (110, 115–117). Additionally, companion dog models have been used for the development of successful cancer drugs including *sunitinib* (cKit inhibitor) for the treatment of renal cell carcinoma and gastrointestinal stromal tumors (110, 118–120) and *ibrutinib* (BTK inhibitor) for the treatment of B-CLL and mantle cell lymphomas (110, 121–123). The clinical stage drugs selinexor (exportin-1 inhibitor) (110, 124) and ganetespib (inhibitor of HSP90 chaperone activity) (110, 125, 126) are currently proceeding through human clinical trials based largely upon successful results observed in outbred dogs.

## Translational Models of Cancer: Patient-Derived Xenografts

Tumors are heterogeneous in their cellular composition, cellular morphology, gene expression, metabolism, cell motility, proliferation, and metastatic potential (127). The tumor microenvironment is complex, and clinically relevant information on tissue context, including cell–cell interactions, or *in situ* variations are lost in *in vitro* studies based on single cell suspensions or tumor cell lines. This critical lack of knowledge is a major obstacle to our understanding of how malignant cells interact with or manipulate the functions of non-malignant surrounding tissue or immune cells, and to the successful development of novel therapies, including immunotherapies (128). However, tumor environments, especially for solid tumors, can largely be preserved in patient-derived xenograft (PDX) models (129). PDX mice (130) are generated by surgical transplantation of small, non-disrupted pieces of primary human lung tumor under the skin of lymphocyte-deficient NOD/SCID/IL2Rγ_c_-KO (NSG) mice.

Athymic (nude) mice, which lack T cells, were first used to generate PDX mice using hematological neoplasms, followed by CB17-*scid* mice, which lack T and B cells. However, it was discovered that NK cells, a cytotoxic immune cell capable of vigorous antitumor responses, are still present in these mouse strains, and that the presence of murine NK successfully restricts tumor growth (131). Thus, current PDX models are generated using lymphopenic (T, B, and NK cell deficient) mice that also harbor defects in innate immunity, such as non-obese-diabetic (NOD)/*scid* and NOD/*scid*/IL-2γ-receptor null (NSG) mice. NSG mice not only have the SIRP1α polymorphism of the NOD mouse, which enables SIRP1a-CD47 interactions that prevent phagocytosis of human cells by murine monocytes (132), but also lack the common gamma chain (IL-2Rγ_c_), resulting in NK cell deficiency and a lack of IL-2, IL-4, IL-7, IL-9, IL-15, and IL-21 signaling (133). Thereby, the use of NSG mice as recipients of human hematological neoplasms, hematopoietic cells and solid tumors, has allowed significant advances in the development of this preclinical model (134, 135).

**Figure 1:**
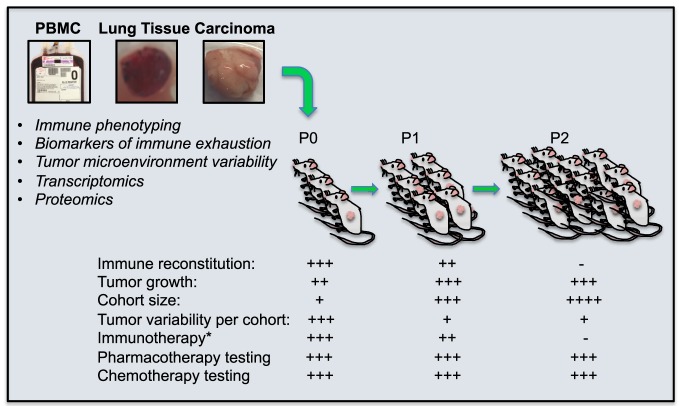
Patient Derived Xenograft (PDX) mice for the evaluation of cancer immunotherapies. PDX mice are generated by surgical transplantation of small, non-disrupted pieces of primary human lung tumor under the skin of lymphocyte-deficient NSG mice. This allows the human tumor to engraft, vascularize, and grow in the immune deficient mice, the periphery of which repopulate with co-transplanted human tumor-associated immune cells that can successfully be targeted with experimental immunotherapy. These immunotherapies can be tailored to tumor-specific pathways of immune exhaustion if immune phenotyping, transcriptomics, or proteomics can be performed on the same tumor. Further, tumors from P0 mice can be excised and these explants transplanted into new NSG hosts (P1). This process can be repeated (P2) to generate ever-larger cohorts of tumor-matched mice. While each PDX transplantation cohort is increased in size, the human immune cells which co-transfer with tumor transplantation, are diluted and eventually lost, abrogating investigative abilities to test immunotherapy approaches that target the endogenous patient-derived immune repertoire. However, pharmacological and chemotherapeutic testing targeting tumor cells directly can still be performed. *endogenous immunotherapy targeting the patients own immune cells.

Tumor transplantation allows the human tumor to engraft, vascularize, and grow in the immune-deficient mice (Figure 1). Tumors from P0 mice can be excised and their explants transplanted into new NSG hosts. While each PDX transplantation round increases the size of the human donor-matched PDX cohort, it also dilutes any co-transferred immune cells and abrogates investigative abilities to test immunotherapy approaches that target the endogenous patient-derived immune repertoire. However, PDX models are nevertheless a valuable translational research tool that enables long-term *in vivo* studies using human tumors in which the tumor heterogeneity and tumor microenvironment has been preserved (129, 135, 136), they enable important preclinical developments of targeted therapeutic strategies, including combination therapies or high-risk strategies, and facilitate bench to bedside transitions (136).

To generate “P0” PDX mice, fresh patient tumors obtained directly from surgery are used to ensure highest tumor cell viability to maximally improve PDX engraftment efficacy (106, 136, 137). The transplantation is either done subcutaneously, into the skin, or orthotopically, into the organ where the original cancer developed. Another, perhaps more complicated method involves tumor tissue transplantation under the murine kidney capsule (136, 138). Ultimately, the goal of all PDX transplantation methods is to allow for the speedy vascularization of the transplanted tumor tissue and its subsequent growth, without the introduction of major changes in the tumor composition or microenvironment. Indeed, a PDX model of gastric cancer recently demonstrated that the engrafted PDX tumor has genetic and histological characteristics highly consistent with the primary tumor (137). In addition to P0 tumors, P1 tumors can be generated from P0 tumors by harvest of the PDX tumor from its murine host, followed by transplantation of P0 PDX tumor explants into non-tumor-bearing recipients. Tumors can thus be maintained by being continuously passaged from mouse to mouse (136). These types of PDX models have successfully been used to study different types of cancer, such as melanoma, breast, pancreatic, ovarian, lung, colorectal, and brain cancer (139). In studies involving PDX models for hepatocellular carcinoma, colorectal cancer, breast cancer, pancreatic ductal adenocarcinoma, head and neck squamous cell carcinoma, adenoid cystic carcinoma, acute lymphoblastic, leukemia, lung, and gastric cancer, the authors concluded that tumors of PDX mice effectively mirrored the histological characteristics, gene-expression, and drug-response features of the corresponding primary tumor.

## PDX Models for the Study of Cancer Immunotherapies

PDX models are also increasingly used to develop and evaluate the efficacy of cancer immunotherapies; however, these studies require the presence of a full repertoire of functional human immune cells (135). For this purpose, researchers have explored ways to humanize both the immune system and the tumor of PDX mice, by co-engrafting the patient tumor fragment with donor-unrelated CD34^+^ human hematopoietic stem cells (HSCs) that are either isolated from human umbilical cord blood, human bone marrow, or human peripheral blood (136, 140): 5 weeks after intrahepatic co-transplantation of human umbilical cord blood-derived CD34^+^ human HSC, and human breast cancer cell lines into the liver of neonatal NSG mice, human immune cells had populated all tissues of the recipient NSG mouse, and tumor cells were detectable in the lungs and bone marrow. Three months posttransplant, tumor-cell effusions, and macroscopic tumors were found in the livers and spleens. Tumor growth was accompanied by the expression of T-cell maturation markers and tumor cell-specific T-cell activation. Importantly, this model can be used to evaluate immunotherapy approaches *in vivo*. In this study, treatments with IL-15/IL-15Rα were designed to stimulate IL-15 receptor expressing cells, such as cytotoxic T cells and NK cells, in trans. Indeed, when both lymphoid and non-lymphoid tissues were examined, the authors reported increased NK cell numbers and NK activation, as well as an expansion of CD3^+^ T cells (both CD4^+^ and CD8^+^ T cells) in non-lymphoid organs (141, 142). Unfortunately, the authors did not report whether this treatment had a significant effect on tumor growth and/or metastasis formation. While the use of CD34^+^ HSC allows for the reconstitution of human innate cells and lymphocytes, and generally does so without the complication of graft-versus-host disease (GVHD), one significant caveat is that all human T cells in this type of xenograft model lack thymic education/proper human leukocyte antigen (HLA)-restriction due to the absence of a human thymus in recipient mice. Similarly, the transplanted human immune system that results from infusions of human CD34^+^ HSC and as such leads to the development of human KIR-expressing NK cells, may or may not be an HLA match to the unrelated human tumor donor. This likely mismatch in HLA expression may further affect NK cell responses to the transplanted tumor (143). Whether this leads to non-physiologic and perhaps difficult to interpret immune interactions within the transplanted tumor remains to be further evaluated.

Alternative ways to reconstitute NSG mice with a human immune system have been evaluated, and one of these approaches is to inject peripheral blood mononuclear cells (PBMCs) into tumor recipient NSG mice to reconstitute the immune system of tumor recipient NSG mice with either donor-unrelated or human-tumor-matched immune cells. Several such studies were published recently and enabled the testing of immunotherapy approaches *in vivo*. We will first discuss results obtained in PDX models that received infusions of human tumor donor unrelated PBMC, followed by a discussion of data obtained in syngeneic PDX models.

In one model, the infusion of peripheral blood lymphocytes and dendritic cells followed by implantation of a human prostate cancer cell line, PC3, resulted in co-engraftment and tumor infiltration by human lymphocytes and enabled an *in vivo* assessment of tumor and immune system interactions (144). In a different model, an orthotopic humanized-xenograft model of human renal clear cell carcinoma (RCC) was generated by the co-implantation of a human RCC cell line into the kidney capsule of NSG mice and simultaneous infusion of human PBMCs that were selected for by high antibody-dependent cytotoxicity activity. The authors chose this approach to evaluate the effectiveness of an antibody specific to the carbonic anhydrase IX protein expressed by the RCC cell line whose Fc portion is capable of binding to and activating NK cells *via* the activating receptor CD16. Antibody immunotherapy lead to tumor infiltration by NK cells and activation of T cells and ultimately resulted in the inhibition of cancer growth (145). While in both cases, co-infusion of human PBMC robustly reconstitutes the human immune system in PDX mice, this approach is clearly only suitable for short-term experiments as it is limited by the rapid onset of GVHD (129), an outcome which may favorably influence antitumor responses in this model and result in exaggerated therapeutic success not easily recapitulated in the clinics, especially when donor-mismatched PMBCs are used.

In a tumor—PBMC donor-matched study, a gastric carcinoma was co-transplanted with syngeneic (tumor donor derived) human PBMC into NSG mice, to evaluate the effects of co-administration of urelumab (anti-hCD137) and nivolumab (anti-hPD-1) *in vivo*. This immunotherapy is designed to simultaneously fight immune exhaustion *via* blocking of the Programmed Death pathway that negatively regulates immune cell antitumor functions (PD-1 blockade with an antagonistic mAb) while simultaneously augmenting immune responses *via* stimulation of 4-1BB (146, 147). In PDX mice in which transferred T lymphocytes expressed the checkpoint inhibitors PD-1 and the tumor necrosis factor family member 4-1BB (hCD137), combination immunotherapy with these two antibodies significantly slowed tumor growth and correlated with the increased activation of IFN-γ-producing human T cells and a decrease in the numbers of human regulatory T lymphocytes in the tumor xenograft (148).

However, to avoid complications related to the infusion of PBMC into mice, a protocol that eventually causes lethal GVHD, improved PDX models that harbor donor-matched tumors and immune cells are needed to improve the physiological relevance of these models for preclinical studies. Theoretically, human donor-matched tumor and immune system reconstitution could be achieved by co-engraftment of tumor tissue as well as bone marrow-derived stem cells, liver, and thymus tissue (131), though this approach would clearly be very invasive for the patient and is thus clinically unacceptable (129). A glimmer of hope came from an early attempt at reconstituting the human immune system of PDX mice with actual tumor-infiltrating co-transferred immune cells. In this model, Simpson-Abelson and colleagues not only demonstrated successful engraftment of solid tumors but also a simultaneous reconstitution of human T cells in NSG mice upon subcutaneous implantation of non-disrupted explants of human primary lung tumor. Several months later, human immune cells were present in the spleen, lung, liver, kidney, and intestine and had an effector memory phenotype. Further, tumor-associated T cells isolated from the spleens of tumor-bearing PDX mice could be maintained and expanded after adoptive transfer into tumor-free NSG recipients (149). These data are encouraging, as a preservation of the tumor microenvironment and the implantation of tumor-associated human donor matched immune cells including HLA-restricted T cells could be achieved, opening the door to target endogenous exhausted immune cells with immunotherapy in the presence of donor-matched tumors.

A similar approach was taken for an ovarian cancer PDX model, in which ovarian tumor cells and tumor stroma, specifically tumor cells and tumor-associated lymphocytes and fibroblasts obtained from patient biopsies, were successfully engrafted into the peritoneum of NSG mice. Encouragingly, the tumor progression in this PDX model mimicked clinically relevant stages observed in ovarian cancer patients: initially, tumor growth was slow in the omentum, ovaries, liver, spleen, uterus, and pancreas, followed by a more rapid tumor growth within the peritoneal cavity, resulting in the occurrence of tumor ascites and spontaneous metastases to the lung. When the authors examined the levels of the ovarian cancer marker CA125 in sera and ascites of PDX mice, they found CA125 levels to increase over time. In addition, both tumor-associated human fibroblasts, and transferred human lymphocytes persisted in this translational ovarian cancer model, and immune cells remained functional, as demonstrated by their ability to respond to cytokine stimulation (150). It is, therefore, not surprising that, despite their limitations, PDX models are now considered preclinical models of cancer, and their use is recommended to monitor overall tumor expression profiles and drug target genes in clinical applications (137, 151).

## Concluding Remarks

Since the beginning of cancer immunotherapy in the nineteenth century, treatment options have evolved to include the use of monoclonal antibodies, immune checkpoint inhibitors, genetically engineered cancer fighting immune cells, cancer vaccines, and combination therapies that combine traditional chemotherapy with one of the above approaches to treat cancer. With the arrival of novel treatment options, a greater need for improved animal and translational models has also emerged. These include highly sophisticated mouse models of cancer, spontaneous cancer models such as the canine model, and translational models bearing transplanted human tumors such as the PDX models. Together, their use will further our understanding of cancer biology and antitumor immunology, allow for a speedier assessment of the efficacy and safety of novel approaches, and ultimately provide a faster bench to beside transition.

## Author Contributions

WD, RS, LA, FG, BB, FK, and SP wrote the manuscript, MS gathered references for the “PDX models for the study of cancer immunotherapies,” section of the article, and SP generated the figure and table.

## Conflict of Interest Statement

The authors declare that the research was conducted in the absence of any commercial or financial relationships that could be construed as a potential conflict of interest.
